# Interference Mechanisms of Endocrine System and Other Systems of Endocrine-Disrupting Chemicals in Cosmetics—In Vitro Studies

**DOI:** 10.1155/ije/2564389

**Published:** 2024-12-03

**Authors:** Yixuan Zhang, Lihong Tu, Jian Chen, Lihong Zhou

**Affiliations:** ^1^NMPA Key Laboratory for Monitoring and Evaluation of Cosmetics, Shanghai Innovation R&D, Testing and Evaluation Technical Service Platform of Cosmetics (22DZ2292100), Department of Evaluation of Cosmetics, Shanghai Municipal Center for Disease Control and Prevention, 1380 Zhongshan Rd. W., Changning, Shanghai 200336, China; ^2^Division of Public Health Service and Safety Assessment, Shanghai Institute of Preventive Medicine, 1380 Zhongshan Rd. W., Changning, Shanghai 200336, China

**Keywords:** cosmetics, endocrine disrupting chemicals, mechanisms, vitro studies

## Abstract

Endocrine-disrupting chemicals (EDCs), found in various cosmetic products, interfere with the normal functioning of the endocrine system, impacting hormone regulation and posing risks to human health. Common cosmetic EDCs, such as ultraviolet (UV) filters, parabens, and triclosan, can enter the human body through different routes, including skin absorption. Their presence has been linked to adverse effects on reproduction, immune function, and development. High-throughput in vitro assays, using various human cell lines, were employed to assess the effects of common cosmetic EDCs such as ethylhexyl methoxycinnamate (EHMC), benzophenone-3 (BP-3), homosalate, and parabens. Despite ongoing regulatory efforts, gaps persist in understanding their long-term impacts, particularly when they are present as mixtures or degradation products in the environment. This study focuses on recent in vitro research to investigate the mechanisms through which cosmetic-related EDCs disrupt the endocrine system and other physiological systems. The in vitro findings highlight the broader systemic impact of these chemicals, extending beyond the endocrine system to include immune, reproductive, and cardiovascular effects. This research underscores the importance of developing safer cosmetic formulations and enhancing public health protection, emphasizing the need for stricter regulations.

## 1. Background

Endocrine-disrupting chemicals (EDCs) are a category of chemical substances altering hormones' homeostasis and interfere with the general functioning of the endocrine system, which in turn influences the development, behavior, and reproduction. They interact with hormone receptors such as the estrogen receptor (ER), androgen receptor (AR), and thyroid hormone receptor (TR) to change the steroid hormone synthesis by inhalation, digestion, and transdermal absorption [1, 2]. EDCs have a lengthy latency period, and exposure to these substances during the stages of prenatal, infancy, and adolescence can cause severe health problems. They can influence the morphology and function of ovaries and uterus, such as steroidogenesis [3, 4], folliculogenesis [4–6] and embryo implantation [7–9]. For children, exposure to EDCs is associated with estradiol (E2), total testosterone (TT), free androgen index (FAI), and TT/E2. Those associations were stronger among pubertal children [10], which suggests a potential effect on proper neurodevelopment. Otherwise, there are positive associations between EDCs and adiposity in boys aged 2.5–4 years [11]. Therefore, the identification and assessment of prevalent EDCs in cosmetics is crucial for safeguarding public health.

The cosmetics sector has emerged as one of the fastest growing industries in the last decade. With increasing public exposure to cosmetics, the safety of cosmetic ingredients has become a major concern in all countries. Some of these cosmetic ingredients are considered EDCs under the European Union CLP (Classification, Labelling and Packaging) regulations. European Chemicals Agency (ECHA) has developed an endocrine disruptor (ED) assessment list including the substances that undergo an ED assessment that have been brought for discussion to ED Expert Group of ECHA. Among these, there are 20 compounds that are related to cosmetic ingredients, such as climbazole, diethyl phthalate, formic acid, and oxybenzone (Table S1). EDCs in cosmetics can enter the body through the skin, mucous membranes, respiratory tract, and digestive tract [12, 13]. Therefore, assessing the endocrine-disrupting effects of cosmetic ingredients has become an important part of ensuring the safety of cosmetic ingredients.

In vitro studies that are based on human cell lines can supply information in ingredients' toxicity studies' early stages and can be combined with in vivo and in vitro studies to construct more comprehensive, scientifically strategies for ingredients' safety testing. A number of assays have been developed with the objective of screening for EDCs in a regulatory context. For instance, the ToxCast and Tox21 assays test thousands of chemicals for endocrine disruption. The US Environmental Protection Agency (US EPA) accepted the results of the ToxCast ER model as an alternative to rodent in vivo assays to screen for estrogenic chemicals. Therefore, in vitro assays are an effective means of screening cosmetic ingredients for endocrine-disrupting properties.

The primary objective of this article is to provide a comprehensive overview of the most prevalent EDCs in cosmetics and observed in vitro studies conducted over the past 5 years. This analysis aims to enhance our understanding of the potential hazards and mechanisms associated with EDCs, while also identifying promising avenues for future research.

## 2. Ultraviolet (UV) Filters

UV filters are substances that are devised to defend our skin from UV-induced harm and can be discovered in many categories of personal care products (PCPs), particularly in sunscreens. The use of sunscreens has increased with the increased awareness regarding the risks of sunburn, photoaging, and skin cancer. UV filters are divided into inorganic UV filters and organic UV filters. Inorganic UV filters commonly used in sunscreen are titanium dioxide and zinc oxide, and the organic UV filters are mainly ethylhexyl methoxycinnamate (EHMC), benzophenone (BP) derivatives, 4-methylbenzylidene methylene camphor (4-MBC), and so on. These UV filters are in almost all water sources around the world and difficult to remove from wastewater using common wastewater treatment plant techniques [14]. Many of these organic UV filters are EDCs, capable of inducing estrogenic activity in in vitro and in vivo test systems [15–18]. In addition, research has shown that they also have antiandrogenic activity and thyroid-disrupting effects [19]. With the gradual deepening of research, the amount of some UV filters added to sunscreen should be carefully considered.

### 2.1. EHMC

EHMC, also known as octyl methoxycinnamate (OMC), is a widely used UV filter in cosmetics, and it has a strong molar extinction coefficient near 305 nm and good formulation suitability, high safety, and large UV absorption coefficients, and is inexpensive. However, the endocrine-disrupting properties of EHMC have been widely reported. EHMC and its metabolites—4-methoxycinnamic acid (4-MCA) and 4′-methoxyacetophenone (4′-MAP), were observed in urine samples from adolescents and Chinese children in the human biomonitoring research, showing an extensive exposure level [20]. EHMC and its metabolite were negatively correlated with stages of testicular volume and genital development [21]. A Chinese study found that childhood EHMC exposure was negatively associated with adiposity measures in peripubertal boys but not girls [22]. In vivo studies showed that the whole body T3 and T4 levels decreased and thyroid hormone-related genes were downregulated after 120-h exposure to EHMC in zebrafish larvae, the vitellogenin gene and thyrotropin releasing hormone were up-regulated, and type II iodothyronine deiodinase was downregulated in Japanese medaka, suggesting that EHMC may affect reproduction and thyroid hormone balance in fish [10, 11]. EHMC exposure is associated with increased infertility due to its antiandrogenic effects, and it also leads to disruptions in gonadotropin-releasing hormone (GnRH) and reproductive hormone levels [19, 23, 24]. These data suggest that EHMC may have endocrine-disrupting effects.

Research studies in recent years have not only focused on the toxic effects of EHMC alone but also focused more on its degradation products and mixture with other contaminants of emerging concern. Lu's research employed the MCF-7-luc to assess the estrogenicity of EHMC-transformation products, which demonstrated a fluctuating estrogenicity with a declining trend [25]. This finding highlighted the potentially greater risks associated with post-transformation cocktails. Cell viability assay and a breast cell line were employed to determine the possible effect of bisphenols and EHMC on human cells. The results showed that EHMC and bisphenols interacted at environmentally relevant concentrations and showed strong synergy or overadditive effects [26]. In the immune system, Ao's study investigated the immunomodulatory effects of EHMC on human macrophages and indicated exposure to EHMC significantly increased the production of various inflammatory cytokines in macrophages, particular tumor necrosis factor-*α* (TNF-*α*), and interleukin-6 (IL-6). In addition, EHMC enhanced the activities of p38 MAPK and the NF-*κ*B signaling pathways, suggesting that EHMC is involved in the regulation of immune-related signaling pathways [27]. In addition, EHMC exposure may be associated with the development and increased risk of cardiovascular disease. Margarida demonstrated that EHMC exposure resulted in the reactivity of the human umbilical artery (HUA) to serotonin and histamine [28]and a rapid and transient relaxation of smooth muscle was observed, which appeared to be mediated by an endothelium-independent mechanism. This effect was comparable to that of estrogens and involved the activation of soluble guanylyl cyclase (sGC), which increased the intracellular levels of cyclic guanosine monophosphate (cGMP) and the inhibition of L-type voltage-operated Ca^2+^ channels (L-type VOCC) [29]. The EHMC altered the contractility patterns of HUA contracted with serotonin, histamine, and KCl. This may be due to an interference with serotonin and histamine receptors or an involvement of the Ca^2+^ channels [30]. The effects and mechanisms of EHMC in vitro studies over the past 5 years are listed in Table 1.

**Figure 1:**
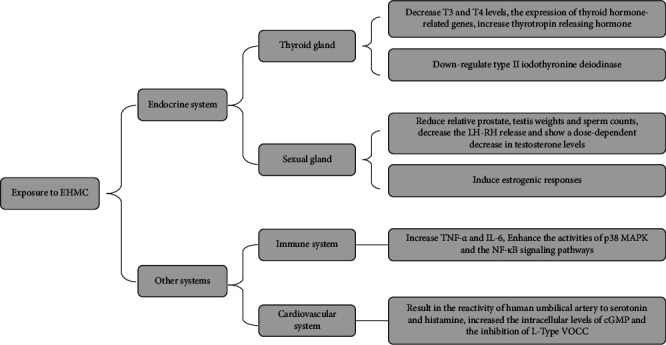
The mechanisms of EHMC in the endocrine system and other systems.

Through the above research results, we found that EHMC can affect the activity of hormone receptors, change the corresponding hormone levels in the body, and cause the endocrine system to dysfunction and have negative effects on health. At the same time, in vitro studies have found that the transformation products of EHMC and mixtures with other EDCs can also produce endocrine-disrupting effects, even stronger than EHMC itself. The impact of EHMC is not only limited to the endocrine system but also affects contractility patterns of HUA and the expression of certain inflammatory factors of the immune system, which can regulate immune-related signaling pathways and the immune function of the body, which provides a new idea for the mechanism of EHMC in the human body (Figure 1).

### 2.2. BP-3

BP-3 is used as a UV filter widely in cosmetics to prevent skin damage and sunburn. BP-3 can be quickly absorbed via oral and dermal paths because it is lipophilic, photostable, and bioaccumulative. However, BP-3 has been found to have multiple endocrine-disrupting effects. Studies have shown that BP-3 exposure leads to hormonal changes in the menstrual cycle and an increased risk of uterine fibroids and endometriosis [43]. Data from the US National Health and Nutrition Examination Survey (NHANES) showed that adult males with elevated urinary BP-3 levels in the United States exhibited a heightened risk of testosterone deficiency and demonstrated a negative correlation with total testosterone, estradiol, and sex hormone-binding globulin levels [44]. These negative effects have made the international community more cautious about the use of BP-3.

BP-3 binds to AR, ER, progesterone receptor (PR), and other nuclear receptors to exhibit endocrine-disrupting properties. Based on a receptor binding assay, weak estrogenic activities and strong antiandrogenic have been shown by BP-3. But at the same time, the antiestrogenic activity is displayed by BP-3 as well [45]. The analysis of binding affinities of BP-3 and its metabolites to sex hormone-binding globulin indirectly indicates that they all possess endocrine-disrupting properties [46]. In recent years, scientists have expanded their research on BP-3 beyond endocrine-disrupting effects and have gained a deeper understanding of the effects of BP-3. As a UV filter, BP-3 comes into direct contact with human skin; therefore, its effects on skin cells were investigated by researchers. BP-3 caused cytotoxicity and induced disturbances in skin cells [31, 32]. BP-3 demonstrated a direct binding affinity for peroxisome proliferator–activated receptor *γ* (PPAR*γ*), resulting in a notable elevation in the transcription of PPAR*α*, PPAR*γ*, and a multitude of lipid metabolism-associated enzymes in human epidermal keratinocytes [33]. Otherwise, Florencia's in vitro experiments revealed cellular adaptive responses that are related to autophagy, lysosomal biogenesis, and ER stress can be affected by BP-3 in pancreatic beta cells, suggesting that BP-3 exposure could result in beta cell dysfunction [34]. In breast cancer, exposure to BP-3 can cause DNA damage and increase migration and invasion of human breast cancer cells [35, 36]. Florence also investigated the bisphenol A (BPA) and BP-3 promoted the T helper (TH)17 cells under TH17-differentiating conditions, while ER *β* expression was decreased, which provided the existence and the immune disruptive potential of real nature EDC mixtures' cumulative effects on T cell solid evidence in vitro differentiation [37]. The effects of BP-3 in vitro studies over the past 5 years are listed in Table 1.

**Figure 2:**
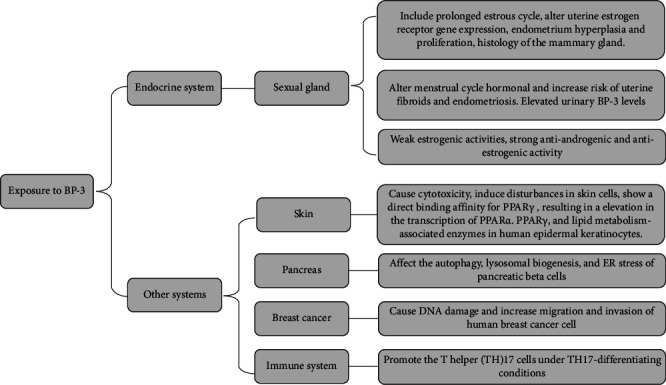
The mechanisms of BP-3 in the endocrine system and other systems.

There have been many in vivo and in vitro studies on the traditional endocrine-disrupting effects of BP-3, while in recent years, scientists have carried out more comprehensive and in-depth studies on the mechanism of action of BP-3 in other systems. BP-3 caused the dysfunction of lipid metabolism and beta cells by binding to PPAR and ER, affecting the development of breast cancer, disrupting the immune system through regulating the differentiation of T cell. For skin cells, BP-3 resulted in some adverse alterations in Type Level I collagen, decorin, sulfated glycosaminoglycans, hyaluronic acid, elastin, and expression or activity of matrix metalloproteinases, elastase, and hyaluronidase, which induced disturbances in skin cells. These in vitro research data of various human systems provide more scientific and rigorous support for the rational use of BP-3 in the future (Figure 2).

### 2.3. Homosalate

Homosalate is used in consumer products mainly as an antioxidant and UV-absorbent, and its residues have been detected in several environmental media. Due to its potential endocrine-disrupting effects, the EU Scientific Committee on Consumer Safety (SCCS) has assessed the safety of homosalate for use in cosmetics. In an assessment opinion issued in June 2021, the SCCS concluded that homosalate esters at a concentration of 10% were no longer safe for consumer health and recommended that the safe concentration be reduced to 0.5%. Subsequently, in its December 2021 reassessment, the SCCS stated that a 7.34% concentration of homosalate is safe for use as a UV filter in creams and facial spray products.

**Figure 3:**
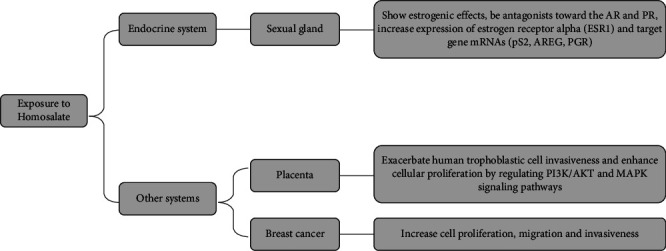
The mechanisms of homosalate in the endocrine system and other systems.

In addition, a study investigated the interfering effects of homosalate on ER, AR, and PR, demonstrating that homosalate showed estrogenic effects and was found to be antagonists toward the AR and PR [47]. Schlumpf's study showed that HMS promoted the cell proliferation of MCF-7 and transactivated ER*α* [38, 48, 49].The researchers evaluated other effects of homosalate using breast cancer cell lines and found that homosalate can increase cell migration and invasiveness in MCF-7 and MDA-MB-231 [35]. Homosalate was found to promote the function of extracellular vesicles (EVs) by significantly increasing the EVs released by triple-negative breast cancer cells, enhancing the resistance of recipient tumor cells to the loss of anchorage and improving their migration ability [39]. The coexposure of polystyrene nanospheres (PNSs) and homosalate resulted in increased expression of estrogen receptor alpha (ESR1) and target gene mRNAs (pS2, AREG, PGR), as well as enhanced transcriptional activation activity of estrogen-responsive elements, which synergistically stimulated the proliferation of MCF-7 cells [40]. Homosalate was found to exacerbate human trophoblastic cell invasiveness and enhance cellular proliferation by regulating PI3K/AKT and MAPK signaling pathways to influence intracellular signaling [41] (Figure 3). The effects of homosalate in vitro studies over the past 5 years are listed in Table 1. These findings suggest the need for more in-depth studies on the safety and potential health risks of homosalate use.

### 2.4. 4-Methylbenzylidene Camphor (4-MBC)

**Figure 4:**
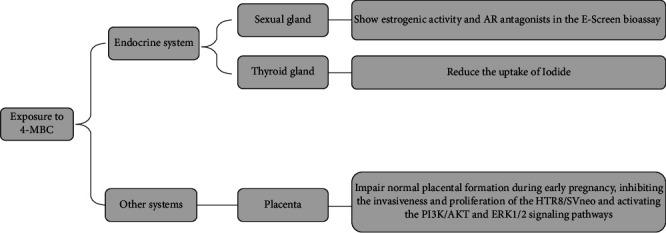
The mechanisms of 4-MBC in the endocrine system and other systems.

4-MBC is an organic UV filter derived from camphor, featuring high stability, which is frequently employed in sunscreens. Research has demonstrated that it disrupted endocrine secretion and led to reproductive difficulties in both vertebrates and invertebrates, as cited in previous reports [50]. 4-MBC promoted the ER-mediated MCF-7 cell proliferation [51–53], showed antagonism of AR and PR transactivation [47, 54], and reduced the uptake of Iodide [55, 56]. It is a teratogen and influences muscular and neuronal development, which may result in developmental defects [57]. Moreover, exposure to 4-MBC inhibited the invasiveness and proliferation of the HTR8/SVneo and activated the PI3K/AKT and ERK1/2 signaling pathways (Figure 4). This may be the mechanism by which 4-MBC impairs normal placental formation during early pregnancy [42]. The effects of 4-MBC in vitro studies over the past 5 years are listed in Table 1.

The use of 4-MBC is decreasing due to its toxic effects. According to a German study, the detection and concentration of 4-MBC metabolites, measurable in the urine of young adults, have significantly decreased to extremely low levels [58]. On April 3, 2024, the European Commission issued Regulation (EU) 2024/996 amending Regulation (EC) 1223/2009 on cosmetic products in the European Union to explicitly prohibit the use of 4-MBC. However, an estimated 15%–20% of UV-filter currently used in China still contain 4-MBC, which may have potential toxic and endocrine-disrupting effects, and, therefore, consumers should urgently seek to address this.

## 3. Preservatives

Preservatives safeguard cosmetic products from microbial contamination, prolonging their shelf life. By preventing possible infections arising from microbe-contaminated cosmetics, the use of preservatives also fosters consumer health. Common types of preservatives comprise formaldehyde-releasing, organic acid, alcohol, and parabens. Among these, parabens are acknowledged as a category of EDCs.

### 3.1. Parabens

Parabens are commonly used as preservatives in cosmetics due to their chemical stability, low cost, low risk of allergic reactions, and broad-spectrum antimicrobial properties. Recent research has revealed that parabens may modulate or disrupt the endocrine system, potentially resulting in adverse effects on human health [59]. These chemicals function as EDCs and are detected in up to 100% of maternal urine samples analyzed [60]. Estimated daily human exposure to parabens from PCPs ranges from 5 to 50 μg/kg/day for adults and 15–230 μg/kg/day for infants and toddlers [61]. A study investigating paraben levels in pregnant women found that higher paraben concentrations were associated with an increased likelihood of preterm birth, shorter gestational age, lower birth weight, and reduced body length at birth [62]. Another study reported that elevated urinary butylparaben (BuP) levels were linked to decreased estradiol levels and a lower estradiol/progesterone ratio in women [63]. Additionally, higher urinary concentrations of methylparaben (MeP) and ethylparaben (EtP) in women were associated with reduced fecundity [64]. A developmental abnormality was discovered by an epidemiological study that examined placenta-specific outcomes in placentas with a positive correlation between placental weight and total maternal urinary paraben levels [65]. Data from the 2007–2008 National Health and Nutrition Examination Survey (USA) revealed that urinary levels of EtP and propylparaben (PrP) were associated with reductions in total T4 levels in both female and male serum samples, as well as decreased free T4 (FT4) levels in female serum. Additionally, serum levels of free triiodothyronine (FT3) were negatively correlated with EtP, PrP, and BuP levels in adult females but not in males [66].

Furthermore, in vitro experiments revealed that parabens interfere with activities of receptors for androgens, estrogens, progesterone, glucocorticoids, and PPARs [67]. BuP exhibited dose-dependent, weak antiandrogenic activity in human breast cancer cells (MDA-kb2), according to Chen's study [68]. Furthermore, MeP, PrP, and BuP at a concentration of 10 μM formed a complex with nuclear receptors, resulting in approximately a 40% reduction in testosterone-induced transcriptional activity [69]. BuP also contributed to decreased expression of CYP19A1, leading to disruptions in both androgen and estrogen levels [70]. Parabens (MeP, EtP, PrP, and BuP) can competitively bind to ERs [71] and exhibit estrogen-like properties. They influence ER-dependent gene expression, affecting proteins such as intestinal calcium–binding protein (ICABP), integral membrane–associated protein-1 (ITMAP1), and calbindin-D9k (CaBP9k) [72, 73]. Current evidence indicates that estrogen disorders are among the factors that trigger the progression of cancer in hormone-sensitive tissues, such as the breast, ovaries, and cervix [74]. Excessive activation of ERs has been shown to upregulate genes responsible for synthesizing growth factors, such as insulin-like growth factor-1 (IGF1) and epidermal growth factor (EGF), both of which promote the growth of cancer cells [75]. Some studies suggest that parabens may bind to nuclear TRs and alter thyroid hormone signaling, although this hypothesis has yet to be confirmed by experimental research [76].

Most studies have focused on a single paraben's effects on estrogen or ARs. However, recent studies most focused on the effects of mixture and mechanisms outside the endocrine system. On the cells of the skin, there is a synergy of cytotoxic activity between MeP and dibutyl phthalate (DBP) [77]. In addition, Arathi's study found that BuP was involved in oxidative stress and the toxicity of BuP observed appeared to be mediated via ATP depletion as seen from luminescence assays [78]. BuP promoted the production of intracellular reactive oxygen species (ROS), induced mitochondrial membrane depolarization, and inhibited the activation of PI3K/AKT pathways in HTR8/SVneo cells, which diminished normal physiological activity of human trophoblast cells [79]. In human lung cells, MeP induced S and G2/M phase arrest and downregulation of mRNA maturation as a result of enhanced apoptosis [80]. Parabens may modulate antiviral immune responses. Lee's study showed that MeP, EtP, and PrP reduced the transcription levels of genes in virus infection-associated pathways, such as IFN-I responses in bone marrow–derived dendritic cells (BMDCs) [81]. The effects of parabens in vitro studies over the past 5 years are listed in Table 2.

**Figure 5:**
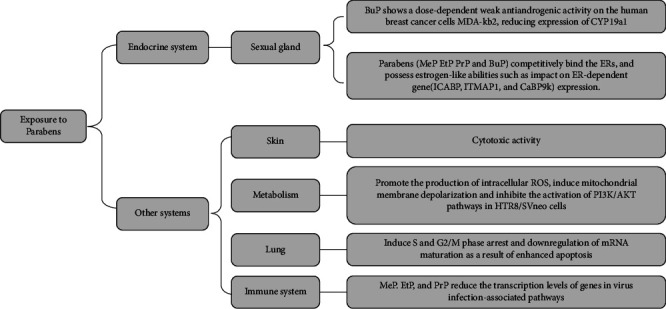
The mechanisms of parabens in the endocrine system and other systems.

Overall, in vitro studies have shown that parabens could disturb the endocrine system by activating the hormone receptors and disrupting the hormone synthesis and secretion, also affect other physiological processes such as apoptosis, oxidative stress, and immune responses by altering gene expression in certain signaling pathways (Figure 5). There is mounting evidence suggesting that parabens may have negative effects on human health, but direct evidence remains scarce. Additional research is required to reconsider the probable health threats of parabens when exposed to realistic level.

### 3.2. Triclosan (TCS)

TCS possesses broad-spectrum antibacterial and antifungal properties and is commonly utilized as a preservative in cosmetic products. It predominantly enters the human body through the skin and mucous membranes but may also enter by inhalation and alternative routes. Its presence can be identified in human urine, blood, breast milk, and amniotic fluid [89, 90]. It contains two phenol functional groups, which suggest its potential to act as an endocrine-disrupting agent. Exposure to TCS has been linked to reduced fecundity, as evidenced by an increased time to achieve pregnancy [91]. The association between TCS exposure and male fecundity was examined in a cohort of couples in Shanghai, China. Elevated TCS concentrations in male urine were linked to reduced fecundability and an increased risk of infertility [92].

In recent years, the effects of TCS on the human body have become a research hotspot, and researchers have used a variety of cellular models to explore the potential health risks and mechanisms of TCS, providing guidance for the rational use of TCS. TCS displaced both estradiol and testosterone from their natural hormone receptors and showed antagonistic activity in both ER- and AR-responsive bioassays, indicating estrogenic and androgenic activity [93, 94]. TCS may act as an agonist of ER in MCF-7 cells to regulate the expression of estrogen-responsive genes (i.e., ERa, pS2) [95]. Forgacs used BLTK1 murine Leydig cells to assess TCS effects, finding that TCS reduced recombinant human chorionic gonadotropin (rhCG)-induced testosterone levels, disrupting the steroidogenic pathway [96, 97]. TCS can disrupt the transcription of P450scc in JEG-3 cells, resulting in estradiol and progesterone production [98].

**Figure 6:**
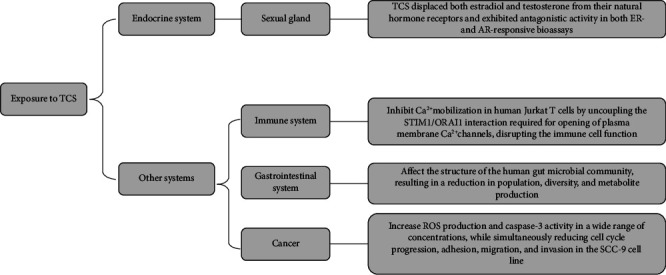
The mechanisms of TCS in the endocrine system and other systems.

It is postulated that oral cancer is caused by genetic factors and exposure to substances derived from cosmetics and disinfectants. TCS is a common contaminant in a wide range of consumer products and oral care products. Studies on oral squamous cell carcinoma showed that TCS increased ROS production and caspase-3 activity in a wide range of concentrations, while simultaneously reducing cell cycle progression, adhesion, migration, and invasion in the SCC-9 cell line [82, 83]. TCS can also enter the gastrointestinal system through the digestive tract. Karley K's research found TCS had a significant impact on the structure of the human gut microbial community, resulting in a reduction in population, diversity, and metabolite production [84]. TCS can also affect the hepatic P450 metabolism. Li's study demonstrated it exhibited significant cytotoxicity on HepG2 expressing hepatic P450 [85]. In addition, Wu's team found that sorafenib resistance is enhanced by TCS treatment in hepatocellular carcinoma (HCC) cells, and this work suggests that the exposure to TCS is detrimental to HCC patients during chemotherapy [86]. For an immune system, Ca^2+^ mobilization was inhibited by TCS in human Jurkat T cells by uncoupling the STIM1/ORAI1 interaction required for opening of plasma membrane Ca^2+^ channels, disrupting the immune cell function [87, 88]. The effects of TCS in vitro studies over the past 5 years are listed in Table 2. Although TCS's mechanism of action has been widely studied (Figure 6), a significantly higher dose is required to achieve toxic effects. TCS has been shown to damage DNA, proteins, and lipids, collectively leading to oxidative stress, necrosis, or apoptosis. In cancer cells, this process is accelerated, resulting in oncogenic transformation's initiation in normal human cells through different receptor pathways. It should be used with caution and strict dosage control.

## 4. Poly- and Perfluoroalkyl Substances (PFASs)

PFAS are synthetic chemicals with multiple fluorine atoms in their alkyl chain. They are used in cosmetics as emulsifiers, antistatic agents, stabilizers, surfactants, and viscosity regulators. These chemicals affect the homeostasis of sex hormones [69] and the thyroid gland function [70]. In China, 45 cosmetic samples were collected and the presence of PFAS was confirmed in all samples, including those products marketed for pregnant women. Perfluorobutanoic acid exhibited the highest placental transfer efficiency among the PFASs detected in cosmetics intended for use by pregnant women [99]. The available evidence indicates that exposure to PFASs in humans is associated with the disruption of the endocrine system, suppression of the immune system, obesity, elevated cholesterol levels, and an increased risk of cancer. It is imperative that these contaminants receive greater attention from health authorities.

The most common PFAS in lotions and nail polishes are perfluorooctanoic acid (PFOA) and perfluorooctane sulfonate (PFOS). In Europe, a new law has limited the use, production, and sale of PFOA and related substances in 2020. The current epidemiological studies linking increased exposure to PFOA/PFOS with lowered testosterone and semen quality [100], lactational exposure to perfluorononanoic acid (PFNA), and PFOS during the postpartum period can exert a negative effect on infant neurodevelopment [101], perfluorohexane sulfonic acid (PFHxS), PFNA, and perfluorodecanoic acid (PFDA) may disrupt androgenic and estrogenic pathways in pregnancy in a sex-dependent manner [102], and exposure to PFOA, PFOS, and PFNA throughout pregnancy is related to a raised danger of preeclampsia and PFOA and PFHxS with gestational hypertension [103].

In vitro studies have demonstrated that PFOA and PFOS exhibit both estrogenic and antiestrogenic effects [104]. PFOS has also been found to alter steroidogenesis [105]. Other studies confirm the estrogenic effects of PFOA and PFOS [106], while some indicate that PFOS is inversely related to estradiol (E2) levels [107]. In H295R cell assays, high concentrations of PFOA and PFOS (10 *μ*M) were associated with increased estrone secretion, and PFOA alone was linked to increased progesterone secretion [108]. Similar results have been observed in other studies using H295R cells [108–110]. PFOA can affect breast tissue development in both mouse models and humans [111]. Research suggests that exposure to per- and polyfluoroalkyl substances (PFASs) may increase breast cancer risk. Using NHANES data, Omoike et al. [112] found that higher levels of PFOA, PFOS, PFNA, PFHxS, and PFDA were linked to increased breast cancer odds. Emerging evidence shows that long- and short-chain PFAS may affect thyroid function in different ways. Both PFOA and HFPO-DA decreased cell viability and proliferation rates in Fisher rat thyroid cells and primary human thyroid cells [113]. Three major classes of emerging PFAS (ePFAS) are chlorinated polyfluoroalkylether sulfonic acids (Cl-PFESAs), hexafluoropropylene oxides (HFPOs), and short-chain perfluoroalkyl acids (SC-PFAAs). These compounds, alternatives to PFOS and PFOA, have similar chemical structures and properties. Cl-PFESA and HFPO have demonstrated higher binding affinity to ERs and ARs than legacy PFAS, interfering with cellular signaling pathways. A number of studies have indicated that ePFAS may have the potential to disrupt endocrine functions and cause hepatotoxicity [114–116].

In addition to the endocrine-disrupting effects discussed above, researchers in recent years have found that other potential effects of PFAS on the human body. Long-chain PFDA and perfluoroundecanoic acid (PFUnA) have been shown to exacerbate degranulation and allergic symptoms in mast cells, in part via their action on the high-affinity IgE receptor (Fc*ε*RI) [117], serving as a factor in allergic inflammation. Furthermore, the interaction of PFOA and PFOS with phase I and II biotransformation enzymes has the potential to result in adverse outcomes, due to the inability of biotransformation pathways to function as required [118]. PFOA also promoted invasiveness of follicular thyroid carcinoma cells (FTC133) mediated through the activation of NF-*κ*B signaling [119]. The effects of PFAS in vitro studies over the past 5 years are listed in Table 3.

**Figure 7:**
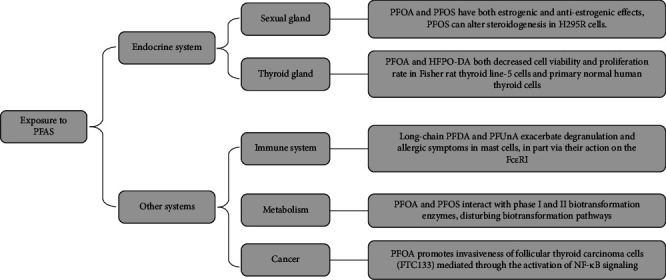
The mechanisms of PFAS in the endocrine system and other systems.

Consequently, the toxicity of PFAS not only is limited to hepatotoxicity and interference with the endocrine system by binding to hormone receptors but also changes gene expression of signaling pathways to regulate lipid metabolism, allergic inflammation, and other physiological activities (Figure 7). These studies provide a clearer understanding of the mechanisms of PFAS and suggest future research directions.

## 5. Microplastics (MPs)

In recent years, MPs have become a commonplace ingredient in cosmetics, functioning as abrasives in a variety of products such as scrubs, exfoliating soaps, body washes, sunscreens, shaving foams, shampoos, and liquid make-up [121]. Since many cosmetic products are applied directly to the skin, and particles smaller than 100 nm can cross the epithelial barrier, the possibility of MPs causing harm to human health through dermal contact cannot be discounted. The size of MPs also affect their absorption through the gastrointestinal tract, alveoli, and skin epithelium [122–124]. Particles measuring 0.1–10 *μ*m have been reported to cross the blood–brain barrier and placenta, while those smaller than 150 μm can traverse the gastrointestinal epithelium. Particles smaller than 2.5 μm enter the systemic circulation via endocytosis [125]. In engineered drug delivery systems, MPs smaller than 5 μm have been observed to accumulate in macrophages and then transported to the mesenteric lymph nodes, circulatory system, and spleen [126]. MPs below the size of 0.1 μm can penetrate cellular membranes, the blood–brain barrier, and even the placenta [127].

Exposure to polystyrene MPs can enhance the likelihood of larger ovaries and fewer follicles in female mice, leading to decreased embryo numbers and reduced pregnancies. Moreover, it alters sex hormone levels and causes oxidative stress, which affects fertility [128]. In men, MPs' detrimental effects comprise abnormal testicular and sperm structure, decreased viability of sperm, and endocrine disruption [129]. These pieces of evidence suggest that MPs have endocrine-disrupting effects and can disrupt the normal functioning of the reproductive system. However, the research studies on the disruption of endocrine function caused by MPs have been limited to correlational and epidemiological studies, and there is a lack of specific in vitro and in vivo experiments to investigate the precise mechanisms for its specific endocrine-disrupting effects.

**Figure 8:**
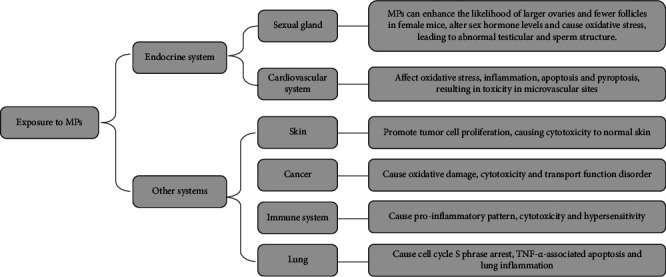
The mechanisms of MPs in the endocrine system and other systems.

MPs have detrimental effects on a wide range of organs and systems in the human body. Studies showed cardiac functions are affected by MPs in humans, resulting in toxicity in microvascular sites. The main underlying mechanisms are oxidative stress, inflammation, apoptosis, pyroptosis, and the interaction between multiple cellular components and MPs [130]. MPs had a high cytotoxicity and caused damage to normal skin but promoted tumor cell proliferation [131, 132], causing oxidative damage and transport function disorder in the human gastrointestinal tract [133, 134]. MP-associated toxicity may be specifically linked to the activation of the ERK pathway, which induces inflammation and oxidative stress ultimately [135, 136]. They can lead to mitochondrial lesions and mitochondria-related apoptosis in the HepG2 cell line and cause hypersensitivity of the immune system [137, 138]. For lung cells, MPs can induce cell cycle S phase arrest and TNF-*α*-associated apoptosis, pulmonary toxicity, and lung inflammation [128, 139, 140]. The effects of MPs in vitro studies over the past 5 years are listed in Table 4. Given the above risks to human health posed by MPs (Figure 8), their use should be strictly regulated.

## 6. Conclusions

With the development of society and the gradual expansion of the cosmetics market, people are becoming increasingly concerned about the safety of cosmetic ingredients. It has been demonstrated that certain ingredients used in the cosmetics have the capacity to exert endocrine-disrupting effects, which can result in a disruption of the normal endocrine function. These ingredients include EHMC, BP-3, homosalate and 4-MBC in sunscreens, certain parabens and TCS in preservatives, and certain PFAS and MPs commonly used in scrubs.

In vitro cell models are convenient to reflect the effects of EDCs quickly and accurately, providing a theoretical basis for subsequent targeted interventions by exploring the mechanism of ED effects through analyses of intracellular nucleic acids and proteins. By analyzing in vitro data from 2018 to 2023, we found that the common EDCs in cosmetics can bind to AR, ER, TR, or other hormone receptors, acting as agonists or antagonists to change the corresponding hormone levels and the expression of the target gene, which result in the dysfunction of the endocrine system and negative effects on health such as premature ovarian aging and developmental disorders. This is the mechanism of major EDs.

In vitro screening methods for specific endocrine mechanisms are designed to determine whether a chemical interferes with a specific endocrine mode of action, based on the specific endocrine-disrupting mechanism and mode of action, for the purpose of screening for the disruptor in question. In vitro methods are simple, rapid and can achieve high-throughput screening. However, due to the lack of in vivo metabolic processes, the endocrine-disrupting activity of metabolites cannot be detected; in addition, these methods mainly target the disruption of the estrogen pathway, the androgen pathway, the thyroid hormone pathway, and the steroidogenic pathway, and the occurrence of a negative result does not rule out the disrupting effect of chemicals through other endocrine pathways, and the occurrence of a positive result only indicates that there is a disrupting activity under a specific endocrine mode of action. Positive results only indicate disruptive activity in a specific endocrine mode of action and do not necessarily result in endocrine-related adverse effects, which need to be further evaluated by in vivo methods. Therefore, assessing the endocrine-disrupting activity of a chemical does require multiple levels of testing, multiple levels of information, and then, a weight-of-evidence analysis before reliable conclusions can be drawn.

In addition, the results of the in vitro studies showed that the impact of EDCs is limited to not only the endocrine system but also other specific organs and systems. They can regulate the expression of certain genes and proteins to cause cytotoxicity, cell cycle abnormality, promotion of tumor cell growth, and effects on the development, function, and lifespan of immune cells, such as monocytes, neutrophils, mast cells, eosinophils, and lymphocytes. EDCs have also been found to increase the incidence of metabolic disorders leading to various adverse effects.

Further studies should focus on the toxicity of EDCs in mixture and should consider its degradation products, to improve the knowledge of its risk assessment as EDCs. At the same time, there are still a large number of cosmetic ingredients that have not been fully evaluated for endocrine disruption and other systemic toxicity. Researchers can use in vitro studies as a convenient and quick way to make an initial assessment of cosmetic ingredients and develop cosmetic production technologies that exclude EDCs, as well as to find harmless, nontoxic alternatives to raw materials in order to minimize human exposure to EDCs. In addition, there is a need to assess the safety of currently used cosmetic ingredients with endocrine-disrupting effects in various aspects and to guide companies to add and use them within safe limits. Relevant authorities must strictly regulate endocrine disrupters in cosmetics, keep track of global trends, and ban the use of harmful substances in a timely manner to protect public health.

## Figures and Tables

**Table 1:** The effects of UV filter in vitro studies over the past 5 years.

EDCs	Model	Effect	References
EHMC-transformation products	MCF-7	Display fluctuating estrogenicity with a declining trend	[25]
EHMC	MCF10A	Show strong synergy or overadditive effects with bisphenols	[26]
EHMC	THP-1	Immune deregulation	[27]
EHMC	HUA	Impair the vascular homeostasis	[28]
EHMC	HUA	Increase the cGMP intracellular levels and an inhibition of L-type VOCC	[29]
EHMC	HUA	Alter the contractility patterns of HUA	[30]
BP-3	BJ	Cytotoxicity	[31]
BP-3	CRL-1474	Induce disturbances in skin cells	[32]
BP-3	hBM-MSCs	Bind to PPAR*γ* and increase the gene transcription of major lipid metabolism-associated enzymes	[33]
BP-3	Pancreatic beta cells	Lead to beta cell dysfunction	[34]
BP-3	MCF-7 and MDA-MB-231	Increase migration and invasion	[35]
BP-3	T47D and MCF-7	DNA damage	[36]
BP-3	Naive T cells	Possess immune disruptive potential	[37]
Homosalate	MCF-7	Genotoxicity	[38]
Homosalate	MCF-7 and MDA-MB-231	Increase migration and invasion	[35]
Homosalate	MDA-MB-231	Increase the EVs released by triple-negative breast cancer cells	[39]
Homosalate	MCF-7	Increase the expression of ESR1, pS2, AREG, PGR and stimulate the proliferation of MCF-7	[40]
Homosalate	HTR8/SVneo	Exacerbate human trophoblastic cell invasiveness and enhance cellular proliferation	[41]
4-MBC	HTR8/SVneo	Retard the normal growth and survival of human trophoblast cells	[42]

**Table 2:** The effects of preservatives in vitro studies over the past 5 years.

EDCs	Model	Effect	References
Methyl paraben	A431	Synergism of action with DBP in cytotoxic effect	[77]
Butyl paraben	HepG2 and HDFn	Cytotoxicity and oxidative stress	[78]
Butyl paraben	HTR8/SVneo	Diminish normal physiological activity of human trophoblast cells	[79]
Methyl paraben	H1299 and MRC5	Cell cycle arrest	[80]
Methyl paraben, ethyl paraben, and propyl paraben	BMDCs	Modulate antiviral immune responses	[81]
TCS	SCC-15	Increase ROS production and caspase-3 activity	[82]
TCS	SCC-9	Reduce cell cycle progression, adhesion, migration, and invasion, induce apoptosis	[83]
TCS	In vitro system of the human gut	Impact the microbial community structure	[84]
TCS	HepG2	Cytotoxic effects	[85]
TCS	MHCC97-H	Enhance sorafenib resistance in HCC cells	[86]
TCS	RBL-2H3, human Jurkat T cells	Disrupt Ca^2+^ influx and immune cell function	[87, 88]

**Table 3:** The effects of PFAS in vitro studies over the past 5 years.

EDCs	Model	Effect	References
Cl-PFESA	MCF7	Promote the proliferation of MCF-7 by activating the ER pathway	[114]
HFPO-DA	JEG3	Downregulate the expression of placental growth factors and genes related to placental transport	[115]
HFPO-DA	FRTL-5	Impair the proliferation of thyroid cells and disrupt the expression of thyroid transcription factor genes	[116]
PFDA and PFUnA	RBL-2H3	Mast cell degranulation and allergic symptoms	[117]
PFOA and PFOS	HepaRG	Reduce I and II biotransformation enzymes	[118]
PFOA	FTC133	Promote invasiveness of FTC133 cells	[119]
PFOS and PFHxS	FRTL-5	Perturb thyroid hormone homeostasis	[120]

**Table 4:** The effects of MPs in vitro studies over the past 5 years.

EDCs	Model	Effect	References
MPs (polystyrene)	SCL-1, A431 and HaCaT	Promote tumor cell proliferation, causing damage to normal skin	[131]
MPs (polystyrene)	HaCaT	High cytotoxicity	[132]
MPs (polystyrene)	HT29	Oxidative damage	[133]
MPs (polystyrene)	Caco-2	Cytotoxicity and transport function disorder	[134]
MPs (polystyrene)	HRT-18 and CMT-93	Pro-inflammatory pattern and cytotoxicity	[135]
MPs (Polytetrafluorethylene)	U937, A549, THP-1,	Oxidative stress and inflammation	[136]
MPs (polystyrene)	HepG2	Mitochondrial lesions and mitochondria-related apoptosis	[137]
MPs (polypropylene)	PBMC, raw 264.7 and HMC-1	Hypersensitivity	[138]
MPs (polystyrene)	A549	Cell cycle S phase arrest and TNF-*α*–associated apoptosis	[139]
MPs (polystyrene)	BEAS-2B	Pulmonary toxicity	[140]
MPs (polystyrene)	MLE12	Lung inflammation	[128]

## Nomenclature

EDCs: Endocrine-disrupting chemicals
ER: Estrogen receptor
AR: Androgen receptor
TR: Thyroid hormone receptor
E2: Estradiol
TT: Total testosterone
FAI: Free androgen index
CLP: Classification, Labelling and Packaging
ECHA: European Chemicals Agency
ED: endocrine disruptor
US EPA: US Environmental Protection Agency
UV: Ultraviolet
PCPs: Personal care products
EHMC: Ethylhexyl methoxycinnamate
BPs: Benzophenone derivatives
4-MBC: 4-methylbenzylidene methylene camphor
4-MCA: 4-methoxycinnamic acid
4′-MAP: 4′-methoxyacetophenone
TNF-α: Tumor necrosis factor-α
IL-6: Interleukin-6
BP-3: Benzophenone-3
BP-1: Benzophenone-1
NHANES: National Health and Nutrition Examination Survey
PR: Progesterone receptor
PPAR: Peroxisome proliferator-activated receptor
BPA: Bisphenol A
TH: T helper
SCCS: Scientific Committee on Consumer Safety
OMC: Octylmethoxycinnamate
HUA: Human umbilical artery
sGC: Soluble guanylyl cyclase
cGMP: Cyclic guanosine monophosphate
L-Type VOCC: L-type voltage-operated Ca^2+^ channels
DBP: Dibutyl phthalate
BMDCs: Bone marrow–derived dendritic cells
TCS: Triclosan
ROS: Reactive oxygen species
HCC: Hepatocellular carcinoma
PFAS: Poly and perfluoroalkyl substances
PFOA: Perfluorooctanoic acid
PFOS: Perfluorooctane sulfonate
PFNA: Perfluorononanoic acid
PFHxS: Perfluorohexane sulfonic acid
PFDA: Perfluorodecanoic acid
Cl-PFESA: Chlorinated polyfluoroalkylether sulfonic acids
HFPO: Hexafluoropropylene oxides
SC-PFAA: Short-chain perfluoroalkyl acids
ePFAS: Emerging PFAS
PFUnA: Perfluoroundecanoic acid
MPs: Microplastics

## Supporting Information

Additional file 1: Table S1. The ECHA endocrine disruptor list of 20 compounds involved in cosmetics.
