# Supplementary material for: Interference Mechanisms of Endocrine System and Other Systems of Endocrine-Disrupting Chemicals in Cosmetics—In Vitro Studies
Source: Int J Endocrinol. 2024 Dec 3;2024:2564389. doi: 10.1155/ije/2564389 (PMC11631346; doi:10.1155/ije/2564389)
Supplement: Supporting Information — Additional supporting information can be found online in the Supporting Information section. [file 2564389.f1.pdf]

Substance name
2,2',6,6'-tetra-tert-butyl-4,4'-methylenediphenol
Climbazole
Diethyl phthalate
Copper
Formic acid
Methyl 4-hydroxybenzoate
Oxybenzone
resorcinol; 1,3-benzenediol
Triclosan
Silver
Isopentyl p-methoxycinnamate
Geraniol
Salicylic acid
Methyl salicylate
Terephthalic acid
Tributyl O-acetylcitrate
p-cresol
butyl 4-hydroxybenzoate
(±)-1,7,7-trimethyl-3-[(4-methylphenyl)methylene]bicyclo[2.2.1]heptan-2-one
Propyl 4-hydroxybenzoate

Status	Outcome
Under development under SEV	Under development (SEV)
Under development under SEV	Under development (SEV)
Under development	Under development (other)
Under development	Under development (BPR)
Under development	Under development (BPR)
Under development under SEV	Under development (SEV)
Under development under SEV	Under development (SEV)
Under development under SEV	Under development (SEV)
Under development under SEV	Under development (SEV)
Under development	Under development (BPR)
Under development under SEV	Under development (SEV)
Under development	Under development (BPR)
Under development	Under development (BPR)
Postponed	postponed
Concluded	not ED
Concluded	not ED
Concluded	not ED
Concluded	ED HH
Concluded	ED HH
Concluded	ED ENV

Application
Commonly used in cosmetics to provide antioxidant and anti-aging benefits.
Preservative, Dandruff Remover
Fragrance fixative
Manufacture of aroma chemicals
Preservative
Preservative
UV-filter
Rinse
Preservative
/
UV-filter
Fragrance
Preservative
Preservative, fragrance
Surfactant
Emulsifiers and stabilizers
Preservative
Preservative
Preservative
Preservative
